# HIV Test Avoidance Among People Who Inject Drugs in Thailand

**DOI:** 10.1007/s10461-012-0347-2

**Published:** 2012-10-23

**Authors:** Lianping Ti, Kanna Hayashi, Karyn Kaplan, Paisan Suwannawong, Evan Wood, Julio Montaner, Thomas Kerr

**Affiliations:** 1 British Columbia Centre for Excellence in HIV/AIDS, St. Paul’s Hospital, 608–1081, Burrard Street, Vancouver, BC V6Z 1Y6 Canada; 2 Interdisciplinary Studies Graduate Program, University of British Columbia, Vancouver, BC Canada; 3 Thai AIDS Treatment Action Group, Bangkok, Thailand; 4 Department of Medicine, University of British Columbia, Vancouver, BC Canada

**Keywords:** HIV testing, Stigma and discrimination, People who inject drugs, Thailand

## Abstract

Case identification is a key component of HIV prevention efforts; yet rates of HIV testing remain low in some settings. We explored factors associated with HIV test avoidance among people who inject drugs (IDU) in Thailand. Between July and October 2011, 350 Thai IDU participated in the study. In bivariate analyses, male gender, high intensity drug use, syringe sharing, increased police presence, and being refused healthcare services were positively associated with HIV test avoidance, while ever receiving a hepatitis C test was negatively associated. Our findings highlight the need for interventions to reduce stigma in this setting.

## Introduction

Thailand has been the site of a longstanding HIV epidemic among people who inject drugs (IDU). Of the estimated 160,000 IDU living in Thailand [1], the prevalence of HIV has consistently remained between 30 and 50 % over two decades [2]. To reduce the health burden of HIV disease among this population, many international health organizations are recommending the scale up of HIV testing services [3, 4]. Previous research has demonstrated that knowledge of HIV-serostatus has been associated with a decrease in high-risk behaviours and can lead to more timely HIV treatment, resulting in reductions in HIV-related morbidity and mortality, as well as HIV transmission [5].

In Thailand, HIV testing services are provided through various means, including in conventional healthcare settings (i.e., hospitals, clinics) and through local and international non-governmental organizations. However, as a result of the ongoing criminalization of illicit drug use due to the 2003 “War on Drugs” campaign and the associated stigma and discrimination IDU often face within conventional healthcare settings [6, 7], some IDU may be reluctant to access HIV testing services. More specifically, the sharing of information on suspected IDU between some hospitals and police may deter these individuals from accessing HIV testing through traditional means [6]. As well, in some voluntary drug treatment centres in Thailand, HIV testing is mandatory prior to receiving services [3]. Consistently with a 2009 United Nations report, on average, approximately 20 % of IDU in the Asia–Pacific region had been previously tested for HIV and were aware of their serostatus in the last 12 months [4].

Though there are some peer-based HIV testing models aimed at reducing the stigma associated with injection drug use and HIV in Thailand, these services are not widespread throughout the country; HIV testing within traditional healthcare settings, where there continues to be high discrimination against IDU, is still considered the conventional route. In an effort to improve the uptake of HIV testing among Thai IDU, we sought to explore the prevalence and correlates of HIV test avoidance among IDU of negative or unknown HIV serostatus in Bangkok, Thailand.

## Methods

Data were derived from the Mitsampan Community Research Project, a collaborative research project involving the Mitsampan Harm Reduction Center (MSHRC) (Bangkok, Thailand), the Thai AIDS Treatment Action Group (Bangkok, Thailand), Chulalongkorn University (Bangkok, Thailand), and the British Columbia Centre for Excellence in HIV/AIDS (Vancouver, Canada). During July and October 2011, 440 IDU residing in Bangkok or adjacent provinces were recruited into a cross-sectional study through peer-based outreach efforts and word-of-mouth. Participants were invited to attend the MSHRC or O-Zone House (drop-in centres for IDU in Bangkok operated by non-governmental organizations) to be part of the study. Participants provided oral informed consent and completed an interviewer-administered questionnaire eliciting information about demographic characteristics, HIV risk and testing behaviour, and other health-related data. Upon completion of the questionnaire, participants received a stipend of 350 Thai Baht (approximately $12 USD). The study was approved by the research ethics boards at Chulalongkorn University and the University of British Columbia.

For the present analyses, we restricted the study sample to individuals who were HIV-negative or of unknown HIV serostatus. We compared the demographic, behavioural, and social/structural characteristics of IDU who avoid HIV testing with those who do not using Pearson X^2^ test. Fisher’s exact test was used when one or more of the cells contained values less than or equal to five. For the purpose of this analysis, HIV test avoidance was measured by asking participants: “Do you sometimes avoid accessing HIV tests because you are a drug user?”. Demographic variables considered included: gender (male vs. female) and median age (≥38 years old vs. <38 years old); behavioural variables included: frequent heroin injection (>weekly vs. ≤weekly), frequent midazolam injection (>weekly vs. ≤weekly), frequent methamphetamine injection (>weekly vs. ≤weekly), binge drug use (yes vs. no), syringe sharing (yes vs. no), unprotected sex (yes vs. no), and ever received a hepatitis C virus (HCV) test; social/structural variables included: ever incarcerated (yes vs. no), ever sent to compulsory drug detention centres (yes vs. no), noticed increased police presence where one buys or uses drugs (yes vs. no), and ever been refused healthcare services (yes vs. no). All variables refer to the previous 6 months unless otherwise indicated. IDU who reported ever being refused healthcare services responded “yes” to the following question: “Have you ever had a healthcare worker refuse to treat you or denied access to medical treatment or care because you are a drug user?” Also, given the high prevalence of HIV and HCV co-infection among IDU [8], and that HIV and HCV services are often delivered together to IDU in many settings, we hypothesized that IDU with a history of HCV testing would be more willing to access HIV services as well. Thus, we included the variable “ever received an HCV test” in our analysis. All *p* values were two-sided.

## Results

In total, 350 IDU with negative or unknown HIV status were recruited into the study, including 68 (19.4 %) females. The median age was 38 years (interquartile range: 34–48 years). In total, 47 (13.4 %) participants reported avoiding HIV testing. In bivariate analyses, as shown in Table 1, avoiding HIV testing was positively associated with a number of demographic and behavioural factors, including: male gender (odds ratio [OR] = 6.27; 95 % confidence interval [CI]: 1.48–26.51), injecting heroin (OR = 2.61; 95 % CI: 1.33–5.13) or midazolam more than once per week (OR = 2.44; 95 % CI: 1.25–4.73), and having lent or borrowed syringes (OR = 2.19; 95 % CI: 1.07–4.47). Ever receiving a hepatitis C virus test was negatively associated with HIV test avoidance (OR = 0.30; 95 % CI: 0.12–0.73). Additionally, the following social/structural factors were associated with HIV test avoidance: noticing increased police presence where one buys or uses drugs (OR = 2.06; 95 % CI: 1.10–3.84), and having been refused treatment or services by health care workers (OR = 6.72; 95 % CI: 3.06–14.74).

**Table 1:** Bivariate analyses of demographic, behavioral, and social/structural factors associated with avoiding HIV testing among a community-recruited IDU in Bangkok, Thailand (*n* = 350)

Avoiding HIV testing				
Characteristic	Yes 47 (13.4 %)	No 303 (86.6 %)	Odds ratio (95 % CI)	*p* value
Demographic variables				
Gender				
Male	45 (95.7)	237 (78.2)	6.27 (1.48–26.51)	<0.01
Female	2 (4.3)	66 (21.8)		
Age				
≥38 years old	24 (51.1)	160 (52.8)	0.93 (0.50–1.72)	0.82
<38 years old	23 (48.9)	143 (47.2)		
Behavioural variables				
Heroin injection^a^				
>weekly	16 (34.0)	50 (16.5)	2.61 (1.33–5.13)	<0.01
≤weekly	31 (66.0)	253 (84.5)		
Midazolam injection^a^				
>weekly	33 (70.2)	149 (49.2)	2.44 (1.25–4.73)	<0.01
≤weekly	14 (29.8)	154 (50.8)		
Methamphetamine injection^a^				
>weekly	6 (12.8)	67 (22.1)	0.52 (0.21–1.27)	0.14
≤weekly	41 (87.2)	236 (77.9)		
Binge use				
Yes	16 (34.0)	89 (29.4)	1.24 (0.65–2.38)	0.52
No	31 (66.0)	214 (70.6)		
Syringe sharing^a^				
Yes	13 (27.7)	45 (14.9)	2.19 (1.07–4.47)	0.03
No	34 (72.3)	258 (85.1)		
Unprotected sex^a^				
Yes	16 (34.0)	107 (35.3)	0.95 (0.49–1.81)	0.87
No	31 (66.0)	196 (64.7)		
Ever received HCV test^b^				
Yes	6 (12.8)	99 (32.9)	0.30 (0.12–0.73)	<0.01
No	41 (87.2)	202 (67.1)		
Social/structural variables				
Ever incarcerated				
Yes	35 (74.5)	215 (71.0)	1.19 (0.59–2.41)	0.62
No	12 (25.5)	88 (29.0)		
Ever sent to compulsory drug detention centers^b^				
Yes	12 (26.1)	56 (18.6)	1.54 (0.75–3.17)	0.23
No	34 (73.9)	245 (81.4)		
Noticed increased police presence where bought or used drugs^a^				
Yes	27 (57.4)	120 (39.6)	2.06 (1.10–3.84)	0.02
No	20 (42.6)	183 (60.4)		
Ever been refused healthcare services				
Yes	14 (29.8)	18 (5.9)	6.72 (3.06–14.74)	<0.01
No	33 (70.2)	285 (94.1)		

*IDU* people who inject drugs, *CI* confidence interval, *HCV* hepatitis C virus

^a^Activities in the previous 6 months

^b^Data does not add up to *n* = 350 due to missing counts

## Discussion

We found that many Thai IDU in our study (13.4 %) reported avoiding HIV testing. Variables significantly and positively associated with HIV test avoidance included a diverse set of demographic, behavioural, and social/structural factors, including: male gender, frequent heroin or midazolam injection, syringe sharing, noticing police presence where drugs are bought or used, and having ever been refused healthcare services. Not surprisingly, IDU who reported ever having received a HCV test were less likely to report avoiding HIV testing.

There are various reasons for why IDU may avoid HIV testing, including: fear of an HIV-positive test result, fear of being stigmatized and discriminated against by the community, and feelings of shame [9]. The fact that Thai public hospitals register information about active drug users may also contribute to the reluctance on the part of IDU to access healthcare [6]. Similarly, prior qualitative research have also indicated comparable reasons for HIV test avoidance among other high risk groups, such as female sex workers and men who have sex with men [10]. Given that to our knowledge, no prior studies have quantified HIV test avoidance among these high risk groups, future research should explore and compare the prevalence of HIV test avoidance among these populations.

A number of demographic and high risk behaviours were found to be associated with HIV test avoidance. We found that males were more likely to avoid HIV testing. This gender difference may reflect the fact that males are overrepresented in the criminal justice system and consequently more likely to avoid testing services out of fear of experiencing discrimination related to their engagement in illegal activities [7, 11]. The finding that participants who avoid HIV testing were also more likely to engage in various high risk behaviours, including high intensity drug use and syringe sharing, is of particular concern. Given that syringe sharing has shown to be a significant route for HIV transmission among IDU, and the high prevalence of HIV among IDU in Thailand [2], novel educational and programmatic efforts are urgently needed to increase rates of testing among this high-risk subpopulation of IDU.

We also found that participants who had ever received HCV testing were less likely to avoid HIV testing. This is reassuring given that high co-infection rates between HCV and HIV have been observed among IDU, with a rate as high as 95 % [8]. However, the fact that more than two-thirds of participants in our study have never received an HCV test is of concern. A previous study conducted on HIV-positive IDU in Bangkok demonstrated that the primary reason for not accessing HCV testing was that the participants had “never heard of HCV” [12]. These findings highlight the need for educational and outreach efforts to increase awareness of and access to both HCV and HIV testing.

The findings from the present study demonstrate that a diverse set of social/structural factors increase the likelihood that IDU will avoid HIV testing. We found that IDU of negative or unknown HIV-serostatus who reported avoiding HIV testing were more likely to report being refused treatment or services from healthcare workers. Previous research has identified that stigmatizing attitudes toward IDU and the outright denial of health services for IDU persists among Thai healthcare professionals, and these attitudes may serve to discourage IDU from further engagement with health systems, including those that provide HIV testing services [7]. As well, noticing increased police presence where one buys or uses drugs was found to be positively associated with avoiding HIV testing. A large body of evidence demonstrates the negative health impacts policing practices can have on the IDU population, yet Thailand continues to rely on aggressive law enforcement approaches for drug control [6]. Interventions that aim to reduce stigma and discrimination against IDU are needed in this setting and other settings like it.

This study has several limitations. First, because of the cross-sectional nature of the study, we cannot determine a temporal relationship between exposure and outcome. Second, the data collected was self-reported and may be subject to reporting biases. Third, since the study sample was not randomly selected, the study findings may not be representative of or generalizable to all Thai IDU or IDU in other settings. Fourth, given that our data was only able to determine whether participants avoided accessing HIV tests due to their drug using behaviour, our analysis was unable to include information on other reasons why IDU may avoid HIV testing. Further, we may have underestimated the total proportion of IDU in our study who avoids HIV testing, as others may avoid testing for other reasons. Lastly, because of the small sample size, there were wide intervals around some of the estimates reported.

In summary, we found a high prevalence of HIV test avoidance among Thai IDU, with males being more likely to report avoiding HIV testing. Various behavioural factors, including binge drug use, syringe sharing, and access to HCV testing, as well as a number of socio-structural factors, including increased police presence and being refused healthcare services by healthcare workers, were also associated with HIV test avoidance. These findings add further evidence regarding the need for ongoing HIV prevention education efforts as well as interventions focused on reducing stigma and discrimination towards IDU.
